# Resistive indices of cerebral arteries in very preterm infants: values throughout stay in the neonatal intensive care unit and impact of patent ductus arteriosus

**DOI:** 10.1007/s00247-016-3615-x

**Published:** 2016-06-03

**Authors:** Ginette M. Ecury-Goossen, Marlou M. A. Raets, Fleur A. Camfferman, Rik H. J. Vos, Joost van Rosmalen, Irwin K. M. Reiss, Paul Govaert, Jeroen Dudink

**Affiliations:** 1 Department of Pediatrics, Division of Neonatology, Erasmus MC-Sophia Children’s Hospital, Wytemaweg 80, 3015 CN Rotterdam, The Netherlands; 2 Department of Pediatrics, Division of Neonatology, Universitair Ziekenhuis Brussel (UZ Brussel), Vrije Universiteit Brussel (VUB), Brussels, Belgium; 3 Department of Biomedical Engineering, Erasmus MC, Rotterdam, The Netherlands; 4 Department of Imaging Physics, Delft University of Technology, Delft, The Netherlands; 5 Department of Biostatistics, Erasmus MC, Rotterdam, The Netherlands; 6 Department of Pediatrics, Koningin Paola Children’s Hospital, Antwerp, Belgium; 7 Department of Radiology, Erasmus MC-Sophia Children’s Hospital, Rotterdam, The Netherlands

**Keywords:** Neonate, Duplex Doppler sonography, Patent ductus arteriosus, Preterm, Resistance index, Resistive index, Ultrasound

## Abstract

**Background:**

Little is known about cerebral artery resistive index values in infants born extremely preterm.

**Objective:**

To report resistive index values in various cerebral arteries in a prospective cohort of preterm infants born at <29 weeks’ gestation, and to compare resistive index in these arteries and assess the relationship between resistive index and hemodynamically significant patent ductus arteriosus.

**Materials and methods:**

Using Doppler imaging, we obtained resistive index values of internal carotid arteries, basilar artery, anterior cerebral artery, and pial and striatal arteries in the first 3 days of age and weekly thereafter until discharge or death. We analyzed paired observations using the Wilcoxon signed-rank test, between-group comparisons with the Mann–Whitney test.

**Results:**

We performed 771 examinations in 235 infants. Resistive indices differed among arteries: vessels with larger diameters showed significantly higher resistive indices. Resistive index in infants without patent ductus arteriosus was lower than that in infants with hemodynamically significant patent ductus arteriosus (median in anterior cerebral artery: 0.75 and 0.82, respectively; *P*<0.001), though this was not statistically significant in all arteries. There was no difference in pre- and post-ligation resistive indices in infants who underwent patent ductus arteriosus ligation.

**Conclusion:**

For accurate follow-up and comparison of cerebral artery resistive index, the same artery should be examined on each occasion.

## Introduction

Cerebral vascular anatomy and disturbance of cerebral hemodynamics are key factors in pathophysiology of brain injury in preterm infants [1]. Therapeutic options for brain injury in these infants are currently lacking, so clinicians focus on prevention through hemodynamic monitoring. Therefore there is renewed interest in noninvasive methods to evaluate cerebral blood flow. One such method assessing one aspect of cerebral blood flow is measuring resistive index in cerebral arteries using color Doppler imaging. The internal carotid artery, basilar artery, anterior cerebral artery and lenticulostriate arteries can be easily visualized with color Doppler imaging [2]. Flow can be evaluated and peak systolic velocity, end-diastolic velocity and resistive index can thus be obtained. Resistive index is defined as (peak systolic velocity – end-diastolic velocity) / peak systolic velocity [3]. In current neonatal clinical practice, resistive index is typically assessed in the anterior cerebral artery in both term and preterm infants admitted to the neonatal intensive care unit. Low resistive index is considered a possible sign of luxury perfusion in term birth asphyxia [3]. A patent ductus arteriosus is considered to be the usual cause for elevated resistive index in preterm infants [3].

Resistive index of cerebral arteries has been described in healthy term neonates [4–10], small groups of clinically stable preterm neonates [9, 11–17] and sick infants [11, 16, 18–20]. These studies included only small numbers of very preterm infants or described resistive index during a short time frame. Results of previous studies in preterm infants are summarized in Table 1. Today more infants born at extremely low gestational age are treated than in prior years, and reference values for resistive index of cerebral arteries in these infants are lacking.

**Table 1:** Resistive index (RI) values in previous studies

Study	Population	Insonated vessels	RI values
Ando 1985 [11]	*n*=14, preterm infants without complications, GA 30–35 weeks	ACA	During first 3 h after birth 0.878 ± 0.057, at 4–6 h 0.855 ± 0.045, at 7–12 h 0.656 ± 0.021
	*n*=13, preterm infants with respiratory distress, GA 25–33 weeks		Results presented in figure
	*n*=8, preterm infants with SEH or IVH, GA 27–33 weeks		No results shown
Blankenberg 1997 [19]	*n*=16, preterm infants, assessed during the first 5–6 days of age, mean GA 26.8 weeks (range 24–31 weeks); 11/16 without PVL or IVH	Lenticulostriate arteries	Mean RI=0.586 ± 0.091
	5/16 developed PVL and/or IVH		Mean RI=0.543 ± 0.087
Calvert 1988 [12]	*n*=29, preterm infants, assessed during the first 72 h of age, GA 25–32 weeks	Right and left cerebral arteries	GA 26–28 RI=0.91 ± 0.09 GA 29–32 RI=0.86 ± 0.08
Evans 1988 [13]	*n*=27, VLBW infants (<1,500 g), assessed during first 7 days of age	ACA	Mean RI=0.79
		MCA	Mean RI=0.81
Lipman 1982 [14]	*n*=8, preterm infants with PDA with left-to-right shunt, GA 26–33 weeks (mean 29 weeks)	ACA	Mean RI=0.90 before ductal closure, 0.76 after ductal closure
	*n*=40, healthy control group, mean GA 31.6 weeks		Mean RI=0.79 (range 0.72–0.85)
Mires 1994 [15]	*n*=137, uncomplicated preterm infants assessed during first 10 days of age 23/137 GA ≤ 32 weeks 44/137 GA 33–34 weeks 70/137 GA ≥35 weeks	ACA and MCA	Results for 3 groups (≤32, 33–34 and >35 weeks) at 1 h and 12 h postnatal age, after 24 h steady state was reached
Pezzati 2002 [9]	*n*=120, healthy neonates, assessed during the first 8 h of age, GA 24–41 weeks	ACA	GA=24–28; RI=0.75 (0.07) ^a^ GA=29–32; RI=0.78 (0.06) ^a^ GA=33–37; RI=0.78 (0.07) ^a^ GA=38–41; RI=0.80 (0.07) ^a^
		MCA bilaterally	GA=24–28; RI=0.76 (0.07) ^a^ GA=29–32; RI=0.80 (0.07) ^a^ GA=33–37; RI=0.82 (0.07) ^a^ GA=38–41; RI=0.81 (0.87) ^a^
Romagnoli 2006 [17]	*n*=70, preterm infants, assessed during the first month of age, mean GA 31.7 weeks (range 25–35) 10/70 GA 25–28 weeks 13/70 GA 29–30 weeks 16/70 GA 31–32 weeks 31/70 GA 33–35 weeks	ACA	- GA=25–28 weeks RI=0.74, 0.71, 0.79, 0.77, 0.76, 0.80 ^b^ - GA=29–30 weeks RI=0.71, 0.75, 0.74, 0.75, 0.78, 0.74 ^b^ - GA=31–32 weeks RI=0.76, 0.74, 0.74, 0.76, 0.78, 0.78 ^b^ - GA=33–35 weeks RI=0.75, 0.75, 0.75, 0.74, 0.76, 0.78 ^b^
		MCA	- GA=25–28 weeks RI=0.75, 0.70, 0.74, 0.76, 0.79, 0.76 ^b^ - GA=29–30 weeks RI=0.73, 0.74, 0.77, 0.77, 0.78, 0.79 ^b^ - GA=31–32 weeks RI=0.73, 0.75, 0.77, 0.77, 0.78, 0.79 ^b^ - GA=33–35 weeks RI=0.74, 0.75, 0.73, 0.77, 0.75, 0.74 ^b^
Seibert 1989 [16]	*n*=57, healthy neonates, mean GA 34 weeks	ACA, MCA and ICA bilaterally	Mean RI=0.75 ± 0.10 in healthy neonates
	*n*=285, ill neonates, GA not mentioned		

*ACA* anterior cerebral artery, *GA* gestational age, *GMH* germinal matrix hemorrhage, *ICA* internal carotid artery, *IVH* intraventricular hemorrhage, *MCA* middle cerebral artery, *PDA* patent ductus arteriosus, *PVL* periventricular leukomalacia, *RI* resistive index, *SEH* subependymal hemorrhage, *VLBW* very low birthweight

^a^ Mean and SD of RI

^b^ 50th percentile of RI values on day 1, 3, 7, 14, 21 and 28 respectively

This study reports resistive index values in various cerebral arteries in a cohort of preterm infants born at <29 weeks of gestation, measured from the first postnatal day throughout their stay in the neonatal intensive care unit. Furthermore, we compare resistive index values among various intracranial arteries, compare resistive index values in left- versus right-side arteries, and assess relationships between resistive index and the Score for Neonatal Acute Physiology, Perinatal Extension, version II (SNAPPE-II scores) [21], gestational age and hemodynamically significant patent ductus arteriosus. We hypothesized that (1) resistive index differs depending on the diameter of the artery where it is measured, such that resistive index is higher in larger intracerebral arteries (e.g., internal carotid arteries, anterior cerebral artery and basilar artery) as compared to smaller intracerebral arteries (e.g., striatal and pial arteries), and (2) that resistive index is higher in the presence of a hemodynamically significant patent ductus arteriosus.

## Materials and methods

The study was approved by the institutional medical ethics review board, and written parental consent was obtained. We included preterm infants born at <29 weeks of gestation admitted to our neonatal intensive care unit between May 2010 and January 2013 for this prospective cohort study. Exclusion criteria included congenital malformation (patent ductus arteriosus was not considered a congenital malformation in this cohort of very preterm infants), parental refusal, and uncertain gestational age. Our department is a tertiary neonatal intensive care unit with an average of 800 admissions per year (including 250–300 very preterm infants).

### Imaging protocol

Infants were examined with cranial US, including color Doppler imaging, according to standard local protocol (on days 0, 1, 2 and 7 after birth and then weekly until discharge or death). Cranial US was performed by two authors with expertise in neonatal cranial US (M.M.A.R. with 4 years of experience in cranial US and P.G. with 25 years of experience). Most of the data were collected by M.M.A.R. In her absence P.G. performed the cranial US.

**Fig. 1:**
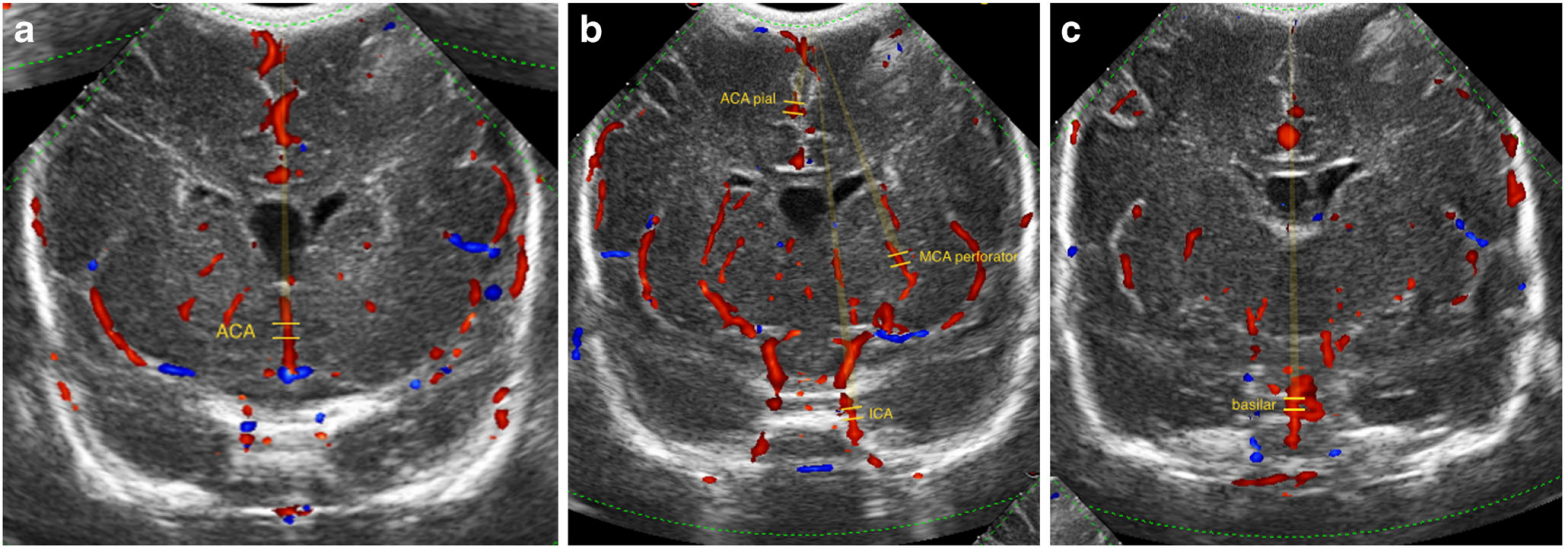
Typical insonation for measurements of Doppler waveforms in this study illustrated in coronal color Doppler transfontanellar (anterior fontanel) US in a 1-month-old preterm girl born at 26 weeks’ gestation, birth weight 900 g. **a-c** Sonograms from anterior (**a**) to posterior (**c**). *ACA* anterior cerebral artery, *ACA pial* pial branch of the anterior cerebral artery, *basilar* basilar artery, *ICA* internal carotid artery, *MCA* middle cerebral artery

Images were obtained in a coronal plane through the anterior fontanel, using the 8.5-MHz convex probe of a MyLab 70 US machine (Esaote, Genova, Italy). A coronal rather than sagittal plane was chosen because in one plane indices can be measured in both large arteries (e.g., basilar artery) and in smaller arteries (e.g., striatal arteries, branches from the middle cerebral artery ascending into putamen). The following intracranial vessels were visualized using color Doppler: internal carotid artery, basilar artery, anterior cerebral artery, striatal arteries and pial arteries (mesial interhemispheric frontal branches of the anterior carotid artery) (Fig. 1). The internal carotid artery and striatal arteries were examined bilaterally. Deviation of protocol occurred on clinical grounds (e.g., hemodynamic or respiratory instability). Doppler settings included pulse repetition frequency of 1.5 kHz, Doppler frequency of 5 MHz, gain of 64%, persistence 16 and depth of 76 mm. Mechanical and thermal indices were kept below 1. Resistive index was manually assessed in the aforementioned arteries using pulsed-wave Doppler. Mean resistive index was calculated from average peak systolic velocity and end-diastolic velocity of at least five sequential stable waveforms. One resistive index assessment per artery was performed on days 0, 1, 2 and 7 after birth and then weekly until discharge or death. Resistive index measurements were performed without knowledge of the clinical condition of the infant. However, it was possible that the observers were aware that ductal ligation had taken place.

We retrieved demographic, perinatal and postnatal data, including SNAPPE-II scores [21], from medical charts and the computerized patient data management system. Patent ductus arteriosus was diagnosed echocardiographically and treated when deemed hemodynamically significant by the investigating cardiologist. Criteria for hemodynamically significant patent ductus arteriosus included ductal size >2 mm, left atrium to aortic root diameter of >1.6, and pulsatile flow pattern of patent ductus arteriosus [22].

### Statistical analysis

This study was part of a large prospective cohort study with the primary objective to investigate structural growth and brain maturation with cranial US in very preterm infants. Because of a lack of similar studies, a proper sample size calculation was not possible. From the onset of the current study it was clear that a larger number of extremely premature infants would be included than in any previous study describing resistive indices in intracranial arteries (see Table 1).

Patient characteristics and resistive index values were described using medians and (interquartile) ranges for continuous variables, and using percentages for categorical variables. Resistive index values were compared between infants with hemodynamically significant patent ductus arteriosus and those without hemodynamically significant patent ductus arteriosus using the Mann–Whitney test (for independent samples). Pairs of resistive index measurements in two arteries from the same subjects were compared with the Wilcoxon signed-rank test (for paired samples). Pairs of pre- and post-ligation resistive index values from the same subjects were also compared using the Wilcoxon signed-rank test. Multivariable linear regression analysis was used to assess the relationship between the independent variables SNAPPE-II score, presence of (hemodynamically significant) patent ductus arteriosus, gestational age and gender, and the continuous dependent variable resistive index. A separate linear regression analysis was performed for each artery. Assessment of the distribution of the residuals in the linear regression analyses was performed using histograms and the Shapiro–Wilk test. Statistical significance was assumed if the two-sided *P*-value was <0.05. Statistical analyses were performed using Microsoft Excel and SPSS (version 21 for Windows; IBM, Armonk, NY).

## Results

During the study period, 333 eligible infants were admitted. We excluded 98 (21 congenital malformation, 3 uncertain gestational age, 12 parental refusal, 62 lack of data [because of death soon after birth, absence of both observers, or insufficient image quality]), leaving 235 to be included. Patient characteristics are shown in Table 2. A total of 771 resistive index examinations were performed in those 235 infants, varying from 1 to 8 (median 3) per subject. This variation is explained by the fact that 38 infants (16%) died while in the neonatal intensive care unit and some infants were transferred to level II hospitals when reaching a corrected age of 30 weeks’ gestation and intensive care was no longer needed. Table 3 lists all resistive index values measured in seven intracranial vessels throughout admission in our neonatal intensive care unit.

**Table 2:** Patient characteristics

	No. (%) or median (range)
Gestational age, weeks	27 (23 6/7 – 28 6/7)
Birth weight, grams	920 (360–1,610)
Male	129 (55%)
Singleton	160 (68%)
Antenatal steroids (doses) none	2 (0–2) 19 (8%)
Vaginal delivery	111 (47%)
SGA	37 (16%)
Apgar score 1 min	6 (0–10)
Apgar score 5 min	8 (1–10)
Umbilical cord pH (*n*=203)	7.31 (6.78–7.48)
SNAPPE II scores (*n*=232)	22 (0–104)
Surfactant (doses)	1 (0–3)
Mechanical ventilation (days)	6 (0–46)
Inotropic support	68 (29%)
PDA (*n*=227)	135 (59%)
Hemodynamically significant PDA	111 (49%)
Treatment of PDA	109/111 (98%)
Medication only	80/109 (73%)
Surgical ligation	29/109 (27%)
Number of days admitted to NICU	31 (1–139)

*PDA* patent ductus arteriosus, *NICU* neonatal intensive care unit, *SGA* small for gestational age, *SNAPPE II* Score for Neonatal Acute Physiology, Perinatal Extension, version II

**Table 3:** Resistive index (RI) values throughout neonatal intensive care unit stay

RI values for corrected age (median [IQR])							
Corrected age (weeks)	ICA right	ICA left	ACA	Striatal right	Striatal left	Pial	Basilar
24	0.80 (0.78-0.87)	0.85 (0.79-0.91)	0.78 (0.72-0.84)	0.61 (0.57-0.66)	0.66 (0.59-0.69)	0.74 (0.67-0.81)	0.80 (0.73-0.85)
25	0.85 (0.74-0.89)	0.85 (0.78-0.91)	0.82 (0.75-0.88)	0.64 (0.60-0.70)	0.68 (0.59-0.71)	0.70 (0.65-0.77)	0.79 (0.74-0.88)
26	0.81 (0.74-0.86)	0.83 (0.78-0.89)	0.81 (0.72-0.86)	0.62 (0.59-0.68)	0.66 (0.60-0.72)	0.67 (0.63-0.73)	0.76 (0.73-0.83)
27	0.84 (0.77-0.89)	0.85 (0.79-0.90)	0.81 (0.74-0.87)	0.64 (0.58-0.70)	0.64 (0.60-0.69)	0.70 (0.66-0.76)	0.79 (0.72-0.85)
28	0.83 (0.77-0.89)	0.84 (0.77-0.89)	0.80 (0.74-0.87)	0.64 (0.58-0.70)	0.64 (0.60-0.69)	0.70 (0.65-0.78)	0.79 (0.73-0.85)
29	0.84 (0.77-0.89)	0.83 (0.77-0.89)	0.80 (0.73-0.85)	0.67 (0.60-0.71)	0.67 (0.60-0.71)	0.71 (0.64-0.78)	0.80 (0.73-0.85)
30	0.84 (0.80-0.88)	0.86 (0.82-0.90)	0.81 (0.77-0.86)	0.66 (0.61-0.71)	0.67 (0.63-0.74)	0.71 (0.68-0.76)	0.82 (0.78-0.86)
31	0.87 (0.80-0.91)	0.86 (0.83-0.92)	0.84 (0.75-0.89)	0.68 (0.65-0.70)	0.66 (0.62-0.70)	0.72 (0.66-0.79)	0.75 (0.74-0.82)
32	0.83 (0.77-0.85)	0.85 (0.79-0.87)	0.80 (0.76-0.85)	0.63 (0.58-0.66)	0.64 (0.63-0.69)	0.72 (0.67-0.74)	0.78 (0.67-0.78)
33	0.89 (0.87-0.91)	0.84 (0.84-0.87)	0.89 (0.88-0.93)	0.60 (0.52-0.64)	0.68 (0.59-0.69)	0.87 (0.86-0.87)	0.81 (0.78-0.82)
34	0.81 (0.78-0.82)	0.79 (0.79-0.82)	0.78 (0.75-0.79)	0.48 (0.48-0.58)	0.53 (0.51-0.60)	0.66 (0.57-0.74)	0.72 (0.72-0.74)
RI values for postnatal age (median [IQR])							
Postnatal age (weeks)	ICA right	ICA left	ACA	Striatal right	Striatal left	Pial	Basilar
0	0.83 (0.76-0.88)	0.84 (0.77-0.89)	0.79 (0.73-0.86)	0.62 (0.57-0.69)	0.64 (0.59-0.69)	0.69 (0.64-0.75)	0.78 (0.71-0.85)
1	0.82 (0.76-0.87)	0.82 (0.78-0.90)	0.81 (0.74-0.85)	0.64 (0.59-0.70)	0.65 (0.60-0.69)	0.70 (0.65-0.77)	0.79 (0.72-0.84)
2	0.84 (0.78-0.90)	0.85 (0.79-0.90)	0.84 (0.75-0.88)	0.66 (0.61-0.72)	0.67 (0.63-0.74)	0.74 (0.68-0.77)	0.80 (0.75-0.87)
3	0.85 (0.80-0.87)	0.86 (0.81-0.91)	0.81 (0.74-0.87)	0.67 (0.63-0.71)	0.65 (0.60-0.70)	0.70 (0.66-0.77)	0.78 (0.74-0.81)
4	0.85 (0.81-0.90)	0.86 (0.77-0.89)	0.82 (0.77-0.88)	0.68 (0.64-0.72)	0.70 (0.63-0.75)	0.77 (0.68-0.80)	0.82 (0.75-0.86)
5	0.83 (0.75-0.89)	0.85 (0.78-0.87)	0.82 (0.76-0.88)	0.64 (0.58-0.68)	0.68 (0.65-0.71)	0.73 (0.68-0.81)	0.86 (0.84-0.86)
6	0.83 (0.77-0.89)	0.88 (0.85-0.90)	0.82 (0.78-0.88)	0.67 (0.63-0.68)	0.64 (0.62-0.68)	0.71 (0.64-0.79)	0.79 (0.72-0.81)
7	0.82 (0.79-0.87)	0.86 (0.84-0.89)	0.79 (0.76-0.83)	0.62 (0.53-0.68)	0.66 (0.50-0.68)	0.74 (0.72-0.78)	0.78 (0.75-0.83)

*ACA* anterior cerebral artery, *ICA* internal carotid artery, *IQR* interquartile range, *RI* resistive index

When comparing resistive index in left- and right-side arteries (measured in the same session in the same subject), resistive index in the left internal carotid artery (median 0.85) was slightly higher than in the right internal carotid artery (median 0.84) (*P*=0.023, sum of ranks 1,824 negative, 3,126 positive). There was no statistically significant difference in resistive index between left- (median 0.64) and right- (median 0.62) side striatal arteries (*P*=0.22, sum of ranks 801 negative, 1,153 positive). Results for comparison of resistive indices in the different arteries (measured in the same session in the same subject) are presented in Table 4. Resistive index in larger arteries (internal carotid arteries, anterior cerebral artery, and basilar artery) was consistently higher than resistive index in the smaller pial and striatal arteries.

**Table 4:** Comparison of resistive index (RI) in different arteries

Comparison	Median RI values	*P-value*	Sum of ranks	
			Negative	Positive
RI ICA right > striatal artery right	0.83 vs. 0.64	<0.001	0	4,095
RI ICA left > striatal artery left	0.84 vs. 0.66	<0.001	2.5	2,554
RI ICA right > pial artery	0.83 vs. 0.70	<0.001	54	2,091
RI ICA left > pial artery	0.84 vs. 0.70	<0.001	12	1,699
RI ACA > striatal artery right	0.81 vs. 0.64	<0.001	2	3,568
RI ACA > striatal artery left	0.81 vs. 0.66	<0.001	3.5	2,275
RI ACA > pial artery	0.81 vs. 0.70	<0.001	41	1,790
RI basilar > striatal artery right	0.79 vs. 0.64	<0.001	26	2,824
RI basilar > striatal artery left	0.79 vs. 0.66	<0.001	7.5	1,589
RI basilar > pial artery	0.79 vs. 0.70	<0.001	134	1,092
RI basilar > ACA	0.79 vs. 0.81	0.126	1,371	2,033

*ACA* anterior cerebral artery, *ICA* internal carotid artery

Ninety-nine infants were diagnosed with focal brain injury at cranial US or MRI at some point during their stay in the neonatal intensive care unit. These focal brain injuries included germinal matrix hemorrhage, intraventricular hemorrhage, periventricular hemorrhagic infarction, cerebellar hemorrhage, perforator stroke and sinovenous thrombosis.

Table 5 presents resistive index values in infants with and without hemodynamically significant patent ductus arteriosus. Nine infants underwent Doppler examination before and after ductal ligation. In four of these, the resistive index in the right internal carotid artery was lower after ligation. Similar results were found in the other six insonated vessels.

**Table 5:** Resistive index values in infants with hemodynamically significant patent ductus arteriosus (PDA) compared to infants without PDA; all values given as median (IQR)

Artery	No PDA	hs-PDA	*P*-*value*	Sum of ranks	
				Negative	Positive
ICA right	0.79 (0.73–0.84)	0.81 (0.75–0.87)	0.11	2.632	3.810
ICA left	0.79 (0.75–0.84)	0.85 (0.80–0.90)	<0.001	1.905	3.766
ACA	0.75 (0.70–0.81)	0.82 (0.78–0.87)	<0.001	2.323	4.698
Striatal right	0.63 (0.58–0.69)	0.65 (0.61–0.70)	0.24	1.765	2.796
Striatal left	0.62 (0.57–0.65)	0.66 (0.59–0.72)	0.06	979	1.948
Pial	0.67 (0.61–0.72)	0.69 (0.66–0.76)	0.03	1.016	1.835
Basilar	0.77 (0.68–0.81)	0.78 (0.73–0.85)	0.046	1.703	2.858

*ACA* anterior cerebral artery, *hs*-*PDA* hemodynamically significant patent ductus arteriosus, *ICA* internal carotid artery, *IQR* interquartile range

Multivariate analysis showed no significant relationship between resistive index and SNAPPE-II score, gestational age or gender. In the anterior cerebral artery and right internal carotid artery there was a significant association between patent ductus arteriosus and resistive index because resistive index in the anterior cerebral artery was 0.069 (95% confidence interval [CI] 0.018–0.120) higher for infants with hemodynamically significant patent ductus arteriosus compared to those without patent ductus arteriosus, and resistive index in the right internal cerebral artery was 0.069 (95% CI 0.023–0.116) higher for infants with hemodynamically significant patent ductus arteriosus compared to those without patent ductus arteriosus. There was no significant association between patent ductus arteriosus and resistive indices in the other five arteries. The residuals in these linear regression models did not have a normal distribution. Therefore we performed bootstrapping to calculate standard errors that are robust to violations of the normality assumption, using the accelerated bias-corrected bootstrapping method. Because the results of bootstrapping are almost identical to those without bootstrapping, we present the results without bootstrapping in Table 6.

**Table 6:** Results linear regression analyses

Artery	Predictor	Coefficient	95**%** CI		*P*-*value*
			Lower bound	Upper bound	
RI ACA	PDA				.030
	no PDA	–.069	–.120	–.018	.008
	PDA, nhs	–.037	–.118	.044	.372
	hs-PDA	Reference			
	SNAPPE score	.001	.000	.003	.080
	GA in weeks	–.006	–.013	.025	.544
	male sex	–.004	–.053	.043	.051
RI ICA right	PDA				.014
	no PDA	–.069	–.116	–.023	.004
	PDA, nhs	–.030	–.101	.041	.398
	hs-PDA	Reference			
	SNAPPE score	.001	.000	.002	.144
	GA in weeks	.007	-.010	.025	.409
	male sex	–.034	-.077	.008	.115
RI ICA left	PDA				.152
	no PDA	–.047	–.096	.003	.063
	PDA, nhs	–.001	–.075	.072	.968
	hs-PDA	Reference			
	SNAPPE score	.001	–.001	.002	.201
	GA in weeks	.002	–.017	.021	.817
	male sex	–.021	–.066	.024	.352
RI striatal artery right	PDA				.152
	no PDA	–.033	–.074	.008	.115
	PDA, nhs	–.050	–.114	.014	.123
	hs-PDA	Reference			
	SNAPPE score	.000	–.001	.002	.659
	GA in weeks	.000	–.015	.015	.962
	male sex	–.005	–.044	.034	.796
RI striatal artery left	PDA				.84
	no PDA	.009	–.027	.044	.633
	PDA, nhs	–.006	–.064	.051	.833
	hs-PDA	Reference			
	SNAPPE score	.000	–.001	.002	.380
	GA in weeks	–.002	–.016	.012	.816
	male sex	–.010	–.044	.024	.561
RI pial artery	PDA				.249
	no PDA	–.050	–.115	.014	.125
	PDA, nhs	–.053	–.146	.039	.254
	hs-PDA	Reference			
	SNAPPE score	.001	–.001	.003	.369
	GA in weeks	.000	–.025	.024	.976
	male sex	–.008	–.068	.052	.788
RI basilar artery	PDA				.323
	no PDA	–.045	–.105	.015	.138
	PDA, nhs	–.028	–.113	.057	.511
	hs-PDA	Reference			
	SNAPPE score	.002	.000	.003	.094
	GA in weeks	.009	–.013	.031	.431
	male sex	–.002	–.055	.051	.937

*ACA* anterior cerebral artery, *CI* confidence interval, *ICA* internal carotid artery, *GA* gestational age, *hs-PDA* hemodynamically significant patent ductus arteriosus, *PDA* patent ductus arteriosus, *PDA*, *nhs* patent ductus arteriosus (not hemodynamically significant), *RI* resistive index, *SNAPPE* Score for Neonatal Acute Physiology, Perinatal Extension

## Discussion

We found that resistive index differed among arteries examined: large arteries showed significantly higher resistive indices than smaller arteries. Thus, when serially assessing resistive index, measurements must be made in the same artery to ensure valid comparison. Infants without patent ductus arteriosus had slightly lower resistive indices than infants with hemodynamically significant patent ductus arteriosus, but this difference did not reach statistical significance in all arteries. In infants who underwent ductal ligation, pre- and post-ligation resistive indices did not differ. We found no significant relationship between resistive index and SNAPPE-II score, gestational age or gender.

In previous work, resistive index in internal carotid artery was higher than in the anterior cerebral artery and middle cerebral artery [16] and lower in lenticulostriate arteries in comparison to the internal carotid artery, anterior cerebral artery and basilar artery [23]. However, in these studies exact resistive index values and statistical significance were not reported. Our study shows that resistive index is related to the diameter of the insonated artery: it was higher in larger arteries. There was a considerable inter-subject difference in resistive index between the larger internal carotid arteries, anterior cerebral artery and basilar artery on one hand, and the smaller pial and striatal arteries on the other hand [24]. This difference can be explained by differences in peak systolic velocity and end-diastolic velocity between the greater and smaller cerebral arteries; in lenticulostriate arteries velocities are lower than in the great cerebral arteries, but the diastolic component is proportionally higher [2]. For accurate follow-up and comparison of resistive indices, it is essential to serially assess the same artery at roughly the same level of its course. With current Doppler US techniques it is not possible to reliably measure the diameter of large intracerebral arteries in extremely preterm infants. Because of this unreliability we did not determine the diameter of the assessed arteries and diameter of the arteries was not included in the linear regression analysis.

In previous studies, there was no difference in resistive index between right- and left-sided arteries [9, 16]. We did not find significant differences in resistive index between left and right striatal arteries in the same subject. However, a slightly higher resistive index was found in left internal carotid artery compared to right. This small difference, although statistically significant, seems clinically irrelevant. It could be a consequence of measurement variability. It might be explained by a ductal steal phenomenon because the duct is situated proximal to the left common carotid artery (and subsequently the left internal carotid artery) and more distal from the brachiocephalic trunk (and thus the right internal carotid artery). The higher resistive index in the left internal carotid artery could be explained by decreased end-diastolic velocity, which could render the left hemisphere more prone to intracerebral lesions, such as stroke or venous infarcts. For example, it has been described that the left hemisphere is preferentially affected in infants with perinatal arterial ischemic stroke [25]. However, stroke is often caused by embolism and it is not straightforward whether arteries with a higher resistive index would be preferentially affected by an embolus. In this study we did not examine the relationship between resistive index and focal brain injury.

In earlier work, resistive index in healthy term and preterm infants was inversely related to gestational age. However, differences related to gestational age were not statistically significant with regression analysis [16]. In a study of 120 healthy preterm and term infants in the first 8 h of age, resistive indices in both the anterior cerebral artery and the middle cerebral artery increased significantly with increasing gestational age [9]. In our cohort of preterm infants born at <29 weeks’ gestation, resistive index was not related to gestational age. This is probably explained by the narrower age range (i.e. 24–29 weeks’ gestation) compared to the aforementioned studies. There was no significant relationship between resistive index and SNAPPE-II score or gender. To our knowledge, there are no studies on the association between resistive index and SNAPPE-II score.

In previous work, an association between patent ductus arteriosus and higher resistive index was attributed to decreased diastolic flow [14]. Therefore it was concluded that cerebral perfusion is affected by patent ductus arteriosus. In the present study, infants without patent ductus arteriosus had lower resistive index values than infants with echocardiographic hemodynamically significant patent ductus arteriosus, but this difference was small and statistically significant in only some of the insonated arteries. Also, resistive index values in these two groups were overlapping for all arteries. This implies that high resistive index values in cerebral arteries are not always indicative of the presence of echocardiographic hemodynamically significant patent ductus arteriosus in very preterm infants. Another possible explanation is that the presence of echocardiographic hemodynamically significant patent ductus arteriosus does not always significantly affect cerebral perfusion, especially in the smaller cerebral arteries such as striatal arteries.

In our cohort, nine infants with echocardiographic hemodynamically significant patent ductus arteriosus underwent Doppler examination before and after ductal ligation. Resistive index was not significantly different after ligation in any of the insonated vessels. In some infants resistive index was lower after ligation, in others it was higher. Two previous studies showed a significant drop in resistive index after ductal closure [14, 26] because of an increase of end-diastolic velocity. Sample sizes were small in both studies, eight and seven infants, respectively.

In our experience, measurement of resistive index is relatively easy and takes little time. Absolute velocity measurements are difficult to compare because they depend on the angle of the probe to the intracerebral vessel [16]. Resistive index value is not affected by changes in probe angle placement, because values for both peak systolic velocity and end-diastolic velocity are affected similarly [7, 16]. It has been shown that assessment of resistive index is reproducible and has high interobserver reliability [2]. However there are some pitfalls. Measurement of resistive index is non-continuous and represents a snapshot in time. As mentioned, resistive index is related to the caliber of the insonated artery. For purposes of comparison and follow-up we therefore recommend to serially assess resistive index in the same artery at roughly the same level of its course. One should be aware that resistive index is an index that does not change when both peak systolic velocity and end-diastolic velocity are similarly affected. For example, resistive index might be normal in a critical situation, such as low blood flow. Resistive index is abnormal only when either peak systolic velocity or end-diastolic velocity is predominantly affected.

For further understanding of the relationship between systemic arterial blood pressure and intracerebral blood flow, cerebral oxygenation and functional state, it would be valuable to simultaneously monitor resistive index combined with invasive arterial blood pressure, regional cerebral saturation and fractional tissue oxygen extraction using near-infrared spectroscopy evaluation of microcirculation [27] and electroencephalogram. Future research might gain insight into the relationship between resistive index and evolution of both unilateral and bilateral focal brain injury in very preterm infants.

Our report has limitations inherent to its design and local logistics. Because infants were transferred to level II hospitals when reaching a corrected age of 30 weeks’ gestation and when intensive care was no longer needed, the number of times resistive index was measured varied from one to eight times per subject. Not all seven arteries were examined in every session, for example when infants showed respiratory instability. One limitation is potential selection bias: because the infants who died soon after birth could not be included in this study, resistive index values in these infants are missing. Although the observers performed the index measurements without knowledge of the clinical condition of the child, it is possible that they were aware that ductal ligation had taken place. Hence the observers were not always blinded. We realize that physiological variables such as PCO_2_ and treatments such as inotropics and blood transfusions could affect cerebral blood flow and consequently resistive index. However, because measurement of variables such as PCO_2_ at the time of cranial US would imply additional blood sampling, we chose not to include these in this study. We did not assess the effect of fontanel pressure on resistive index. All measurements were made while fontanel pressure was kept to a minimum by applying a generous amount of gel, and low pressure was confirmed by routinely visualizing flow through the superior sagittal sinus.

## Conclusion

Resistive indices differed depending on the caliber of the examined artery: larger vessels showed significantly higher resistive indices than smaller vessels. Therefore, for accurate follow-up and comparison of resistive index of cerebral arteries in these infants, it is important to examine the same artery at roughly the same level of its course. Infants without patent ductus arteriosus had a slightly lower resistive index than infants with hemodynamically significant patent ductus arteriosus, but this difference did not reach statistical significance in all arteries. In infants who underwent ductal ligation, pre- and post-ligation resistive indices did not differ.
